# Defending legitimate epidemiologic research: combating Lysenko pseudoscience

**DOI:** 10.1186/1742-5573-4-11

**Published:** 2007-10-10

**Authors:** James E Enstrom

**Affiliations:** 1 University of California, Los Angeles, CA, USA; 2 Scientific Integrity Institute, Los Angeles, CA, USA

## Abstract

This analysis presents a detailed defense of my epidemiologic research in the May 17, 2003 *British Medical Journal *that found no significant relationship between environmental tobacco smoke (ETS) and tobacco-related mortality. In order to defend the honesty and scientific integrity of my research, I have identified and addressed in a detailed manner several unethical and erroneous attacks on this research. Specifically, I have demonstrated that this research is not "fatally flawed," that I have not made "inappropriate use" of the underlying database, and that my findings agree with other United States results on this relationship. My research suggests, contrary to popular claims, that there is not a causal relationship between ETS and mortality in the U.S. responsible for 50,000 excess annual deaths, but rather there is a weak and inconsistent relationship. The popular claims tend to damage the credibility of epidemiology.

In addition, I address the omission of my research from the 2006 Surgeon General's Report on Involuntary Smoking and the inclusion of it in a massive U.S. Department of Justice racketeering lawsuit. I refute erroneous statements made by powerful U.S. epidemiologists and activists about me and my research and I defend the funding used to conduct this research. Finally, I compare many aspect of ETS epidemiology in the U.S. with pseudoscience in the Soviet Union during the period of Trofim Denisovich Lysenko. Overall, this paper is intended to defend legitimate research against illegitimate criticism by those who have attempted to suppress and discredit it because it does not support their ideological and political agendas. Hopefully, this defense will help other scientists defend their legitimate research and combat "Lysenko pseudoscience."

## Background

This analysis presents a detailed response to the extensive attacks that have been made on my epidemiologic research in the May 17, 2003 *British Medical Journal*, "Environmental tobacco smoke and tobacco related mortality in a prospective study of Californians during 1960–98" [1]. I seek to defend the honesty and scientific integrity of my research and I directly respond to my most powerful critics, who have attempted to suppress and discredit findings that do not support their ideological and political agendas. To put a historical perspective on the tactics that have been used against me, I conclude by making an analogy with the pseudoscientific practices of Trofim Denisovich Lysenko [2]. Hopefully, my defense will encourage and/or help other honest scientists to defend their research against unwarranted and illegitimate criticism.

This analysis deals with several important elements of the attacks, with a primary focus on the epidemiologic issues involved. Additional elements of the attack are mentioned briefly in this analysis and are presented in detail on my Scientific Integrity Institute website, under 'Research Defense' [3]. Being attacked for publishing unpopular scientific findings is not unique to me or my research. However, the nature and scope of the attacks to which I have been subjected is quite unusual and needs to be documented and addressed.

Being able to distinguish between real and implied scientific misconduct is important to the integrity of science in general and to the integrity of individual scientists in particular. Falsely accusing an honest scientist of scientific misconduct is just as wrong as scientific misconduct itself. Implying that an honest scientist has committed scientific misconduct because he has published unpopular findings or has used an unpopular funding source is wrong and falls under the category of "scientific McCarthyism" [4].

## Analysis

### Background on *BMJ *Paper

I begin with a presentation of the background necessary to understand the issues involved with the May 17, 2003 *British Medical Journal *(*BMJ*) paper that I wrote with Dr. Geoffrey C. Kabat [1]. This account primarily involves me and thus is written in the first person, but it also refers to Kabat where appropriate and not otherwise noted. Our paper found no relationship between environmental tobacco smoke (ETS) and tobacco-related mortality in a prospective study of Californians during 1960–1998, with some associations slightly below the null and some slightly above the null, but none statistically different from the null. It concluded, "The association between exposure to environmental tobacco smoke and coronary heart disease and lung cancer may be considerably weaker than generally believed." It is the largest (in terms of statistical power), most detailed (in terms of results presented), and most transparent (in terms of information about its conduct) epidemiologic paper on ETS and mortality ever published in a major medical journal.

The study is based on the California (CA) portion of the original 25-state Cancer Prevention Study (CPS I) [1]. CA CPS I was begun by the American Cancer Society (ACS) in 1959 and has been conducted at UCLA by me since 1991. Kabat and I are both well qualified epidemiologists who have had long and successful careers dating back to the 1970s, as can be confirmed by examining our epidemiologic publications on PubMed. Our paper was deemed to be scientifically sound and worthy of publication after being peer reviewed by two distinguished epidemiologists, a *BMJ *statistician, and a *BMJ *editorial committee. The details of the entire peer review process and the names of all the individuals involved in the review process are available online as the "Prepublication history" [5]. The paper was subjected to the same review process and selection criteria as other papers submitted to the *BMJ*, which publishes less than 10% of the total submissions it receives [6].

In the interest of transparency and full disclosure, the paper included the following detailed statements about the funding history of the study and the competing interests of the authors: "Funding: The American Cancer Society initiated CPS I in 1959, conducted follow up until 1972, and has maintained the original database. Extended follow up until 1997 was conducted at the University of California at Los Angeles with initial support from the Tobacco-Related Disease Research Program, a University of California research organisation funded by the Proposition 99 cigarette surtax. After continuing support from the Tobacco-Related Disease Research Program was denied, follow up through 1999 and data analysis were conducted at University of California at Los Angeles with support from the Center for Indoor Air Research, a 1988–99 research organisation that received funding primarily from US tobacco companies. Competing interests: In recent years JEE has received funds originating from the tobacco industry for his tobacco related epidemiological research because it has been impossible for him to obtain equivalent funds from other sources. GCK never received funds originating from the tobacco industry until last year, when he conducted an epidemiological review for a law firm which has several tobacco companies as clients. He has served as a consultant to the University of California at Los Angeles for this paper. JEE and GCK have no other competing interests. They are both lifelong non-smokers whose primary interest is an accurate determination of the health effects of tobacco." [1].

### Initial Attacks on *BMJ *paper

Even though our paper satisfied (and in many ways exceeded) the accepted standards of epidemiologic analysis and writing, it was immediately attacked by people who did not like the results we reported. Beginning in the days before May 17, 2003, our *BMJ *paper was subjected to a large-scale *ad hominem *attack. Since our honesty or scientific integrity had never previously been questioned, such an attack seemed to us to be quite implausible and indeed incredible. Based on what I have learned since May 2003, I describe the key elements of this attack in order to expose the tactics that have been used in an attempt to discredit and silence legitimate epidemiologic research. Additional details are presented on my Scientific Integrity Institute website [3]. The attack has been largely due to the fact that we published politically incorrect null findings from a long-term study primarily funded by the ACS, but completed with a research award to UCLA from the Center for Indoor Air Research (CIAR), a now-defunct tobacco-industry funded research organization.

On May 9, 2003 I learned that our paper was to be published in the May 17, 2003 *BMJ *and that an embargoed *BMJ *press release was to be issued on May 13, 2003. The strict publication/broadcast embargo regarding our paper was to last until 00:01 hours (UK time) on May 16, 2003, which was 19:01 (7:01 PM) EDT on May 15, 2003 in Florida and 16:01 (4:01 PM) PDT on May 15, 2003 in California. During this period, the ACS was informed of our forthcoming paper and the press embargo. The ACS then prepared its own press release entitled "American Cancer Society Condemns Tobacco Industry Study for Inaccurate Use of Data." The May 14, 2003 version of the ACS press release was inserted into a May 15, 2003 email message of Stanton A. Glantz, Ph.D., Professor of Medicine at the University of California, San Francisco (UCSF). Glantz send out this message worldwide to his UCSF listserv before the press embargo ended [7]. The official May 15, 2003 version of the ACS press release, which adhered to the press embargo, was issued in a separate PDF form [8]. Then it was permanently posted on the ACS web site in a slightly different format [9].

The instantaneous attack on our paper appears to have been a coordinated effort, primarily organized by the ACS and Glantz. Glantz is a well-known anti-smoking activist who has worked closely with the ACS for many years [10]. As part of this coordinated effort, Glantz organized a May 15, 2003 Miami, Florida press conference involving a panel of "international experts" in order to "debunk" our "Marry a Smoker, Get Less Cancer" study before the press embargo ended [11]. At the time of the ACS press release and the Miami press conference, neither the ACS, Glantz, or the other Miami "experts" had access to the full ten-page version of our paper, let alone time to read it and carefully analyze it. The full version of our paper was not posted on the *BMJ *website until the press embargo lifted at 7:01 PM EDT on May 15, 2003 [1]. The only version available when the embargoed *BMJ *press release was issued on May 13, 2003 was the abridged five-page paper that appears in the print version of the *BMJ *[12]. Obviously, these critics chose to hastily write a press release and hold a press conference based on limited information. They did not have the integrity or objectivity to read our full ten-page paper or to contact the authors before beginning their attack, which included erroneous claims about the paper's content and quality.

The ACS press release was authored by Michael J. Thun, M.D., ACS Vice President, Epidemiology and Surveillance Research, and Harmon J. Eyre, M.D., ACS Chief Medical Officer. This press release makes several entirely false statements about the study, such as:

1) "Tobacco Industry Study" was "Part of Organized Effort to Confuse Public About Secondhand Smoke"

2) "Society researchers repeatedly advised Dr. Enstrom that using CPS-I data to study the effects of secondhand smoke would lead to unreliable results"

3) "this study is neither reliable nor independent"

4) "The study suffers from a critical design flaw: the inability to distinguish people who were exposed to secondhand smoke from those who were not"

5) "exposure to secondhand smoke was so pervasive [in 1959] that virtually everyone was exposed to ETS, whether or not they were married to a smoker".

Further distracting from the actual content of the study and the legitimacy of the analysis, the press release added a number of out of context quotes from formerly confidential tobacco industry documents that had nothing to do with the conduct, analysis, or publication of the study. For the past several years these documents have been available online from the Legacy Tobacco Documents Library at UCSF [13], which was established by Glantz [14]. These documents are also available at other online tobacco document libraries [15]. As shown above, my tobacco industry funding and competing interests were clearly and accurately described in more than 200 words in the *BMJ *paper [1]. However, in order to raise doubts about my honesty and scientific integrity, the ACS made a great effort to locate and extract selective quotes from the professional correspondence I have had with the tobacco industry during my career. This *ad hominem *attack diverted attention from the paper itself and obscured its contribution to the body of epidemiologic evidence regarding the lethality of ETS.

A major element of the attack included the submission to the *BMJ *website of over 150 mostly negative electronic letters, known as "rapid responses" (rrs) [16]. The overall content and nature of these rrs was summarized by a *BMJ *associate editor in an August 30, 2003 letter [17]. Particularly troubling are May 19 and 20, 2003 rrs by Thun [18,19], a May 30, 2003 rr by Thun and 13 other members of the International Agency for Research on Cancer (IARC) Working Group on tobacco smoke [20], and a August 19, 2003 rr by Drs. Phillip S. Gardiner, Charles Gruder, and Francisco Buchting of the University of California Office of the President [21]. None of the authors of these criticisms ever contacted us for a clarification of any aspect of our *BMJ *paper or our contacts with the tobacco industry before posting their rrs.

Most of the press coverage of the study was muted or equivocal because of the issues raised by the ACS criticism of the paper. Typical of this type of newspaper coverage was the May 16, 2003 Los Angeles Times article on page A26, "Study Downplays the Health Risks From Secondhand Smoke." This article concludes with the following quote from Dr. Jonathan Samet, Professor and Chair of Epidemiology at the Johns Hopkins University Bloomberg School of Public Health: "We have one very flawed study that does not find an association. It flies in the face of so much evidence and so much scientific understanding that it just doesn't contribute." [22].

### Supportive Commentary on the *BMJ *Paper

A supportive press account appeared in the May 18, 2003 *Sunday Telegraph *newspaper article, "Warning: the health police can seriously addle your brain," by Robert Matthews [23]. The article noted, "More than any other health debate, the question of whether smokers kill others as well as themselves is engulfed in a smog of political correctness and dubious science." Other supportive commentaries also appeared. Michael Fumento, a Senior Fellow at the Hudson Institute, wrote a September 11, 2003 syndicated column, "Second-hand Smoke is Harmful to Science" [24]. Elizabeth Whelan, Sc.D., President of the American Council on Science and Health (ACSH), wrote an August 13, 2004 ACSH column entitled "American Cancer Society a Danger to Science?" [25]. Michael Fitzpatrick, M.D., a general practice physician in London, wrote a November 15, 2004 *Spiked *commentary entitled "We have ways of making you stop smoking." [26]. These commentaries put our *BMJ *findings in context and described the excesses of the anti-smoking critics who attacked us.

Two sociologists, Drs. Sheldon Ungar and Dennis Bray, noticed the rrs and the other media coverage of my paper and described the phenomena that they observed in their own January 2005 paper [27]. They described in detail the "efforts to prevent the making of specific scientific claims in any *or *all of the arenas in which these claims are typically reported or circulated" as they related to my *BMJ *paper. Their "results suggest that the public consensus about the negative effects of passive smoke is so strong that it has become part of a regime of truth that cannot be intelligibly questioned." Given all the controversies involving other epidemiologic risk factors, such as, hormone replacement therapy, air pollution, and vitamin supplements, this state of affairs regarding ETS is quite amazing. Indeed, the evidence regarding the lethality of ETS is not "a regime of truth," but collection of weak results that have turned into a "causal" relationship by carefully chosen committees. As I will discuss later, the epidemiologic evidence on this subject has changed in recent years and needs to be completely and objectively reassessed in order to reach a valid conclusion.

### Authors and Editor Defend the *BMJ *Paper

The attack described above was quite startling to me as someone whose honesty and scientific integrity had never been questioned during the 33-year period from July 1970, when I received my Ph.D. [28], until May 2003 [1]. It was also startling that the attack was initiated by the ACS, the very organization that had given me the original California Cancer Prevention Study (CA CPS I) data in 1991 upon which the *BMJ *study was based. Kabat and I dealt with some of the initial controversy by responding to specific criticisms in our August 30, 2003 *BMJ *letter [29] and in our January 31, 2004 *Lancet *letter [30]. In particular, in these letters we refuted the five false ACS statements shown above:

1) This was not a "Tobacco Industry Study," but rather a UCLA study conducted by two qualified epidemiologists with ACS cooperation up until publication of the *BMJ *paper. This was not "Part of Organized Effort to Confuse Public About Secondhand Smoke", but rather it was an accurate representation of the results of one study. The tobacco industry played no role in the conduct, writing, or publication of the paper, and did not even know it was being published until it appeared.

2) It is a complete fabrication that "Society researchers repeatedly advised Dr. Enstrom that using CPS-I data to study the effects of secondhand smoke would lead to unreliable results." Indeed, the ACS Vice President for Epidemiology prior to Thun worked closely with me on the overall CA CPS I follow-up study from 1991 until 2001 because he felt that this was a valuable project. He was a co-author on the first version of the ETS and mortality paper when it was submitted to the *New England Journal of Medicine *in 2001 and was co-author on my first publication based on the CA CPS I cohort, which dealt with smoking cessation and mortality trends [31]. He was not able to remain as co-author on the ETS and mortality paper after 2001 because of his retirement from the ACS and his growing distance from the project.

3) It is absolutely false that "this study is neither reliable nor independent." First, this study is just as reliable as other epidemiological studies that have been conducted in a similar manner Indeed, the *BMJ *peer review process found that the results of the study were sound and sufficiently reliable to be worthy of publication and the ACS has thus far identified no specific errors in the study. Second, the study was conducted independent of influence from both the ACS and the tobacco industry.

4) It is absolutely false that "The study suffers from a critical design flaw: the inability to distinguish people who were exposed to secondhand smoke from those who were not." This cohort study was done in the same way as the other spousal smoking studies and our 1999 follow-up questionnaire survey results clearly showed that there were subjects who had varying degrees of exposure to ETS as shown in Tables 1 and 2 of the *BMJ *paper. This issue was clearly addressed in the *BMJ *paper in response to Thun's 1999 concerns about this issue [32].

5) It is absolutely false that "exposure to secondhand smoke was so pervasive [in 1959] that virtually everyone was exposed to ETS, whether or not they were married to a smoker." The results of the 1999 survey shown in Table 4 of the *BMJ *paper clearly showed that among never smokers married to never smokers as of 1959, 43.5% of males and 61.7% of females reported no regular exposure to cigarette smoke from others in work or daily life as of 1999.

Although the ACS disputes the validity of my 1999 survey, they have not conducted their own ETS exposure survey of the approximately 50 million Americans who were born before 1950 and who are currently alive. Such a survey would yield actual evidence as to whether or not all Americans alive during the 1950s and 1960s were equally exposed to ETS. The ACS cannot simply make an unsubstantiated claim that "virtually everyone was exposed to ETS" and expect this claim to negate all the evidence presented in my *BMJ *paper.

In addition to the published letters cited above, we submitted to the *BMJ *on June 30, 2003 Manuscript BMJ/2003/084269, a detailed commentary that vigorously defended specific aspects of our *BMJ *paper. We showed that there was, in fact, substantial agreement between our results regarding ETS and those of the ACS and pointed out inconsistencies in ACS findings that had not been previously noted. Unfortunately, on September 19, 2003 the *BMJ *declined to publish this commentary, which would have helped resolve the controversy that had erupted over our *BMJ *paper. We then spent over two years attempting to publish various portions of this commentary in other journals until we successfully published in 2006, as described in our January 24, 2006 rr to bmj.com [33]. Portions of Manuscript BMJ/2003/084269 are presented later in this paper and the entire manuscript is posted for historical reference [34].

In spite of the numerous attacks described above, the *BMJ *has stood behind the *BMJ *paper since its publication. For all of the vehemence of the rrs, only about 3% referred to actual data in the paper and none identified anything approaching scientific error or scientific fraud [16]. Indeed, our paper was ranked among the "Top tens from bmj.com" in 2003 [3531]. *BMJ *Editor Richard Smith strongly defended his decision to publish the paper on both May 18, 2003 [36] and August 30, 2003 [37]. Furthermore, Smith again defended this decision in his 2006 book, *The Trouble with Medical Journals*, in which he stated "it would be antiscience to suppress systematically one source of research" [38]. To date, no impropriety, bias, or omission has been identified in the review process and no error in the results has been identified in the paper, not even by Thun, who is in a position to check our findings and to publish additional findings.

### Support for the *BMJ *paper from Other Epidemiologic Research

To further document the validity of our *BMJ *findings, Kabat and I compared them with the other U.S. epidemiologic evidence on ETS and coronary heart disease (CHD), in our 2006 peer-reviewed meta-analysis of environmental tobacco smoke and CHD mortality in the United States [39]. This comprehensive meta-analysis focuses on the U.S. cohort studies of ETS and CHD death in never smokers. These cohort studies are all fairly similar in design; ETS exposure was approximated by spousal smoking; CHD death was the endpoint; and they constitute virtually all the U.S. evidence and the majority of the world-wide evidence. In contrast to the previous major meta-analyses on this topic, such as the one in 1999 by Thun [32], our analysis includes the results of our 2003 study and the 1995 study by LeVois and Layard based on CPS I data [40]. We have applied consistent criteria to the selection of results included in the analysis. The results are summarized in terms of overall relative risks and dose-response relationships. In addition, available data on misclassification of ETS exposure, personal monitoring of actual ETS exposure, and dose-response data for active smoking are discussed in order to characterize the estimates of ETS exposure in epidemiologic studies.

Contrary to the claims of the ACS and other critics, our results do not differ in any material way from those of the other studies, particularly for females. A further example of the ACS misrepresentations on the ETS issue can be found in the following simple comparison of statements about the findings in their major 1982 Cancer Prevention Study (CPS II) cohort. In the May 15, 2003 ACS press release Harmon J. Eyre, MD, stated: "CPS-II is one of more than 50 studies now published that have shown non-smokers married to smokers have an increased risk of lung cancer" [8,9]. But, the 1995 doctoral dissertation based on CPS II by Victor Cardenas, "*Environmental tobacco smoke and lung cancer mortality in the American Cancer Society's Cancer Prevention Study II*", was inconclusive [41]. The dissertation abstract states: "This study found no evidence of an association between self-reported ETS and lung cancer risk among nonsmokers. However, using spousal smoking habits to assess exposure, we found ETS is only weakly, and not statistically significantly, related to lung cancer risk among nonsmoking women in seven years of follow-up of the CPS II cohort." [41]. Even though our findings are entirely consistent with Cardenas' findings, Eyre impugned our study with his statement: "Bad science can haunt us for generations. And regrettably, if questionable studies make it to publication, the damage is done." [8,9].

Furthermore, we specifically refuted the unsubstantiated claim by Thun that our *BMJ *study is "fatally flawed because of misclassification of exposure" [42]. Thun implied that virtually everyone in the U.S. during the 1950s and 1960s was equally exposed to ETS because it was so pervasive. Results from four independent surveys, as well as our 1999 CA CPS I survey, show that Americans were not equally exposed to ETS. Additional surveys show that exposure to ETS comes primarily from spousal smoking, not public smoking, particularly for females. Indeed, there was a clear relationship between spousal smoking and self-reported ETS exposure among never smokers who lived a major portion of their life before the introduction of restrictions on public smoking in the 1970s. One of these surveys is contained in the 1995 Cardenas dissertation [41]. Although Thun served on the Cardenas dissertation committee, to my knowledge, he has never cited results from this dissertation.

We found that when all relevant studies are included in the meta-analysis and the results of the individual studies are appropriately combined, current or ever exposure to ETS, as approximated by spousal smoking, is associated with roughly a 5% increased risk of death from CHD in never smokers, not the widely cited 25% in the meta-analyses of Thun and others. Furthermore, we found no dose-response relationship and no elevated risk associated with the highest level of ETS exposure in males or females.

Another paper which sheds light on the CPS II findings concerning ETS is a 1995 analysis which linked data on ambient air pollution from 151 U.S. metropolitan areas with mortality data from CPS II individuals who resided in those areas [43]. The results of this analysis showed that in never smokers there was a statistically significant association of all cause mortality with both sulfate and fine particle concentrations after controlling for covariates, including *"hours per day of ETS exposure*." The authors, one of whom was Thun, did not report the specific results for the confounding variable of ETS exposure. However, in order to resolve a major dispute over the validity of the results in this air pollution analysis [44], a reanalysis was conducted in 2000 by the Health Effects Institute (HEI) [45]. The Cox proportional hazards regression model (PHREG) results included in Appendix F of the resulting HEI Reanalysis Report make it clear that the independent variable "passive" (hours per day of ETS exposure) shows no association with mortality from lung cancer, cardiopulmonary disease, or all causes in never smokers [46]. Results are shown as a relative risk (RR) and 95% confidence interval (CI). For lung cancer in CPS II, RR(passive) = 1.020 (0.938–1.110) for males, 1.004 (0.995–1.013) for females, and 1.005 (0.957–1.055) for both sexes. These relative risks agree well with those in my CA CPS I study, where RR(7 level index) = 0.88 (0.70–1.10) in males and RR(8 level index) = 0.97 (0.90–1.05) in females. For cardiopulmonary diseases in CPS II, RR(passive) = 1.004 (0.987–1.021) for males, 1.015 (1.000–1.029) for females, and 1.010 (0.999–1.021) for both sexes. For all causes in CPS II, RR(passive) = 0.996 (0.984–1.009) for males, 1.004 (0.995–1.013) for females, and 1.001 (0.994–1.009) for both sexes. A key portion of the actual PHREG computer printout for these diseases for males, females, and both sexes has been assembled and posted [46]. The PHREG program used in the CPS II study [43,46] is the same as that used in the CA CPS I study [1].

My *BMJ *results for coronary heart disease are also consistent with those in the Western New York State study published in the October 9, 2006 *Archives of Internal Medicine*, which found "After adjustment for covariates, exposure to secondhand smoke [SHS] was not significantly associated with an increased risk of myocardial infarction [MI]" [47]. Furthermore, this study concluded "Exposure to SHS has declined sharply among nonsmokers in recent years. In the absence of high levels of recent exposure to SHS, cumulative lifetime exposure to SHS may not be as important a risk factor for MI as previously thought." This study was entirely independent of my study and was done without tobacco industry funding and came to the same conclusion with regard to heart disease. Finally, my *BMJ *results for lung cancer in the CA CPS I cohort are consistent with those of the original 1981 ACS analysis of the nationwide CPS I cohort [48]. This analysis examined lung cancer mortality during 1960–1972 and found "Compared with nonsmoking women married to nonsmoking husbands, nonsmokers married to smoking husbands showed very little, if any, increased risk of lung cancer." This analysis was entirely funded by and conducted by ACS and came to the same conclusion as my *BMJ *analysis.

### Ongoing Misrepresentations Regarding ETS

Much of the evidence above is not being properly presented and there is misrepresentation of other evidence. For instance, serious misrepresentation of CPS II results is evident when one examines the 1997 Cardenas peer-reviewed paper [49], which was based on the 1995 Cardenas dissertation [41]. Table 4 of the Cardenas paper presents exposure to spousal smoking among women by the husband's level of smoking, but is deceptively labeled. Women with the highest level of exposure, labeled "40+ cpd by spouse", have a RR of 1.9 (95% CI 1.0–3.6) and the P for dose-response trend is 0.03 (cpd = cigarettes per day). However, Table 38 of the Cardenas dissertation makes clear that the RR for spouses of *current *smokers of 40+ cpd is only 0.9 (95% CI 0.2–3.9) and the P for trend is 0.34. If it were not for Table 38 the reader would not know that Table 4 is based on the combination of current and former smokers. This combination of current and former smokers by cpd is highly unorthodox, has not been done in other ETS studies, and is not meaningful for assessing a trend based on current spousal smoking. The Cardenas dissertation makes it very clear that there is no dose-response relationship between spousal smoking and lung cancer in CPS II. Key sections of Cardenas' Tables 4 and 38 are shown side by side in Table 1 and they reveal a serious discrepancy in the presentation of the same data. Because Cardenas' Table 38 appears to present the underlying findings and because these findings contradict Eyre's statement above, the ACS should clarify this major discrepancy. However, no clarification has been made and only the positive dose-response relationship in Cardenas' Table 4 is ever cited [49].

**Table 1:** Comparison of the CPS II dose-response results of 1995 Cardenas disseration and 1997 Cardenas paper: relative risk (RR & 95% CI) of lung cancer death by ETS exposure (spousal smoking) among female never smokers in CPS II. Definition in 1995 Cardenas dissertation [41]: 'Analyses restricted to nonsmoking spouses married to nonsmoking spouses and those married to cigarette smokers (and not other type of tobacco), with complete smoking data, married once at a time of interview, and with valid data on age at first marriage.' Definition in1997 Cardenas paper [49]: 'The referent group includes never-smoking women married to husbands who did not smoke during the marriage. The exposed categories are split into approximate tertiles, and are restricted to never-smokers married to cigarette smokers with complete smoking data, married once, and with valid information on age at marriage.'

1995 Cardenas dissertation [41]					1997 Cardenas paper [49]		
	
Spousal smoking (cigarettes per day)	Deaths/Person-years	1982–89 CPS II Fully-adjusted RR (95% CI)	Deaths/Person-years	1982–89 CPS II Fully-adjusted RR (95% CI)	Cigarettes per day by spouse	Deaths/Person-years	1982–89 CPS II Fully-adjusted RR (95% CI)
				
	Table 38 as shown on page 117		Proper summary of Table 38 data		Table 4: improper summary of Table 38 data		
			
Never	30/311,333	1.0	30/311,333	1.0	0 (never)	30/333,946	1.0
Former(1–19)	4/61,677	0.6 (0.2–1.8)					
Former(20–39)	12/120,585	0.8 (0.4–1.7)					
Former(40+)	11/49,304	2.0 (1.0–4.0)					
Former – total			27/231,566	1.13 (0.72–1.78)			
Current(1–19)	5/32,524	1.7 (0.7–4.4)	5/32,524	1.7 (0.7–4.4)	1–19 (current or former)	9/83,074*	1.1 (0.5–2.2)
Current(20–39)	10/69,060	1.6 (0.8–3.4)	10/69,060	1.6 (0.8–3.4)	20–39 (current or former)	22/179,751*	1.2 (0.7–2.2)
Current(40+)	2/24,900	0.9 (0.2–3.9)	2/24,900	0.9 (0.2–3.9)	40+ (current or former)	13/71,618*	1.9 (1.0–3.6)
P test for trend for 'former'		P = 0.29					
P test for trend for 'current'		P = 0.34			P test for 'current or former'		P = 0.03

* Current and Former Combined

For instance, Cardenas' Table 4 findings are now cited in the 2004 WHO IARC Monograph 83 "Tobacco Smoke and Involuntary Smoking" [50]. This major 1452-page report contains a review of the epidemiologic evidence on ETS and lung cancer on pages 1231–1271 [51]. The section "Exposure-response relationships" on page 1236 contains the statement "The study by Cardenas *et al*. (1997) also found a significant exposure-response relationship. When the husbands smoked 1–19, 20–39, ≥40 cigarettes/day, the relative risks for women exposed to secondhand smoke were 1.1, 1.2, and 1.9 respectively (p value for trend test, 0.03)".

In addition, a January 2004 *J Natl Cancer Inst (JNCI)*summary of IARC Monograph 83 shows results for ≥40 cigarettes/day in Table 3 and it contains the erroneous value RR = 1.9 [52]. Obviously Thun, a member of the IARC Working Group for Monograph 83, did not notify the IARC Working Group about the 1995 Cardenas dissertation. This type of selective analysis and presentation of results has been termed "publication bias *in situ*" and it is often difficult to detect [53]. I was able to detect this irregularity only because I knew of the Cardenas dissertation. In other scientific fields, the type of data manipulation done in Cardenas' Table 4 would most likely be treated as a serious ethical violation. Also, it is noteworthy that 14 authors of the *JNCI *article signed an August 30, 2003 *BMJ *letter criticizing my *BMJ *paper, but then made no mention of my paper in their January 2004 *JNCI *article.

### Continuing ACS Campaign to Discredit the *BMJ *Study

Although I have refuted the erroneous statements in their May 15, 2003 press release, the ACS has shown no interest in correcting the record with regard to me and my research. Their press release has been posted on up to 1,000 locations on the Internet during the past four years, based on Google searches of the phrase "American Cancer Society Condemns Tobacco Industry Study." It is still posted on many websites in addition to ACS's own website. Our *BMJ *and *Lancet *letters and our new meta-analysis defending the validity of our *BMJ *paper are being ignored by the ACS. Instead, the ACS and other activist organizations continue to post defamatory information about us and our research.

Our new meta-analysis shows that the relationship between ETS and CHD in U.S. never smokers is very weak (estimated relative risk of 1.05 with no dose-response relationship) [39]. Yet the ACS continues to state in their 2007 "Cancer Facts and Figures" that "ETS causes an estimated 35,000 deaths from heart disease in persons who are not current smokers" (page 36) [54]. The source the ACS uses for this CHD death estimate is a 1992 *JAMA *paper [55], even though more than 90% of the U.S. epidemiologic evidence has been published since 1992. Our new meta-analysis shows that the vast majority of the existing U.S. evidence originates from the ACS CPS I and CPS II cohorts, yet the ACS simply ignores or dismisses most of this evidence. The CPS I and CPS II evidence is summarized in Table 2, which is taken from Table 6 of our meta-analysis paper [39].

**Table 2:** Dose-response relationship between ETS exposure and CHD mortality. Relative risk of spousal smoking related to CHD deaths among never smokers in CA CPS I [1], CPS II [82], and CPS I [40] and in the summary RR of the three studies.

Spousal smoking	1960–98 CA CPS I Age-adjusted RR (95% CI)	1982–89 CPS II Fully-adjusted RR (95% CI)	1960–72 CPS I Age-adjusted RR (95% CI)	Summmary 'Age-adjusted' RR (95% CI)
				
	Enstrom [1] (extracted from Tables 7 & 8)	Steenland [82](extracted from Table 2)	LeVois [40] (extracted from Table 4)	Enstrom + Steenland + LeVois
Males				
Never	1.00	1.00	1.00	1.00
Former	0.94 (0.78–1.12)	0.96 (0.83–1.11)	0.95 (0.83–1.09)	0.95 (0.87–1.04)
current				
1–19 cigs/day	0.91* (0.78–1.06)	1.33 (1.09–1.61)	0.99 (0.89–1.09)	1.02 (0.94–1.10)
20 cigs/day	0.92 (0.74–1.15)	1.17 (0.92–1.48)		
20+ cigs/day			0.96* (0.83–1.11)	1.02 (0.92–1.12)
21+ cigs/day	1.20* (0.88–1.64)	1.09 (0.77–1.53)		
				
Females				
Never	1.00	1.00	1.00	1.00
Former	1.02 (0.93–1.11)	1.00 (0.88–1.13)	0.99 (0.93–1.05)	1.00 (0.95–1.05)
Current				
1–19 cigs/day	1.07* (0.96–1.19)	1.15 (0.90–1.48)	1.04 (0.97–1.12)	1.05 (0.99–1.12)
20 cigs/day	1.04 (0.92–1.16)	1.07 (0.83–1.40)		
20–39 cigs/day			1.06 (0.98–1.15)	1.04 (0.98–1.10)
21–39 cigs/day	0.95 (0.80–1.12)	0.99 (0.67–1.47)		
40+ cigs/day	0.83 (0.65–1.06)	1.04 (0.67–1.61)	0.95 (0.78–1.15)	0.92 (0.79–1.06)

* indicates RR was based on combining other RRs

### Continuing Glantz Campaign to Discredit Enstrom

Beginning with his activities at the time of the publication of our *BMJ *paper, Glantz has continually attacked me and my research, in spite of the fact that we are both established, long-term faculty members in the University of California system. Glantz is well-known as a long-time anti-smoking activist [10,56], whose ultimate goal is achieving a society free of smokers [57]. However, as a UC faculty member, he is supposed to adhere to the UCSF Campus Code of Conduct [58] and the UC Standards of Ethical Conduct [59]. For instance, the Code of Conduct states "Misconduct or Misconduct in Science means fabrication, falsification, plagiarism, or other practices that seriously deviate from those that are commonly accepted within the scientific community for proposing, conducting, or reporting research." The UC Standards of Ethical Conduct states "Members of the University community are expected to conduct themselves ethically, honestly, and with integrity in all dealings."

However, based on his clearly documented written and verbal attack on me, he has not adhered to these codes. Indeed, I have spent the past four years responding to his false and misleading statements and defending my honesty and scientific integrity. The full details of his campaign are too extensive to present here, but the selected examples below demonstrate the tactics that he used against me and the epidemiologic research that I have been conducting at UCLA.

On July 25, 2003 Neal L. Benowitz, MD, UCSF Professor of Medicine, and Glantz co-wrote an eight-page letter to the UC Vice Provost for Research Lawrence Coleman which attempts to make the case that acceptance of tobacco industry funding for research violates current Regents and University policy and should be ended [60]. On pages 3 and 4 of this letter they claim: "The most recent example of how the tobacco industry uses funding of university research as part of its for propaganda campaign is a May 17, 2003 study from UCLA on the health effects of secondhand smoke published in the *British Medical Journal*. . . . There is little possibility that it will be taken seriously in scientific circles. . . . this paper would go down as one bit of poor research done at a university with a reputation for high quality scholarship that slipped into a good journal because of the foibles of the peer review process."

On March 8, 2005 Glantz participated with other UC faculty members in a San Francisco based KQED radio program entitled "Funders and Academic Research: *Forum *assesses the controversy surrounding the relationship between funders and academic research," which can be listened to on the Internet and audio files [61]. During this program Glantz attempted to discredit well qualified scientists and their peer-reviewed research publications by inappropriately linking them to the tobacco industry. The "scandal" about me and my *BMJ *study was discussed during minutes 17–19 of this 52 minute program, when Glantz made several clearly false and inflammatory statements. First, Glantz claimed that the *BMJ *study "was not funded by the American Cancer Society," but was "done with Philip Morris' money." Actually, the study was funded by ACS from 1959 to 1990, by the UC Tobacco-Related Disease Research Program from 1991 to 1997, and by the Center for Indoor Air Research (CIAR) from 1998 to 2003. Philip Morris provided no direct funding for this study and had no role in its conduct. Then, Glantz stated that I was "a damn fool" who was told by ACS that I "made inappropriate use of the data", an unsubstantiated claim made only after Glantz and ACS learned of my results. Then, Glantz implied that I was "advocating a pro-tobacco position" when I have never done so. Finally, Glantz claimed "the science that the UCLA study did was crap", whereas it clearly conformed to the standards of epidemiologic research. These statements indicate the unprofessional approach used by Glantz to attack scientific findings with which he disagrees and to advocate positions that are not supported by the facts.

Glantz's arguments for banning tobacco industry funding of research at UC have been rejected in favor of academic freedom. The UC administration has expressed its strong support for academic freedom and UC Vice Provost for Research Coleman has stated "Academic freedom must be absolute or no one has it" [62]. On May 11, 2005 the UC Academic Senate adopted a strong Academic Senate Resolution on Research Funding Sources which clearly supports the right of individual UC faculty members to accept research support from any source, including the tobacco industry, as long as this funding adheres to University policy [63]. In spite of this strong faculty resolution, in September 2006 Glantz brought the issue of a ban on tobacco industry funding to the UC Regents, the governing body of the University [64]. Glantz cited my *BMJ *study as one rationale for such a ban in written documents [65] and in a January 18, 2007 presentation before the UC Regents [66]. The UC Regents requested advice on this issue from the UC Academic Senate, which spent several months carefully evaluating the matter [67]. My perspective, including a defense of my research, my funding, and my scientific integrity, was presented to the UC Academic Senate in April 2007 [68]. In May 2007 representatives of the UC Academic Senate voted almost unanimously (15 to 1 by the Academic Council and 44 to 5 by the Academic Assembly) in favor in academic freedom and against a proposed ban on tobacco industry funding advocated by Glantz [69-71].

One final example of Glantz's unprofessional treatment of my research is contained in his May 24, 2005 *Circulation *report, where he attempts to make the case that passive smoking has nearly the same impact as active smoking on cardiovascular effects [72]. In his meta-analysis of the relation between ETS and CHD, he found "The pooled relative risk computed with a random-effects model (computed with Stata Version 7) was 1.31 (95% CI, 1.21 to 1.41), similar to the estimates of earlier meta-analyses." To achieve this result, he omitted the two largest studies, which represent a major portion of the available evidence. My *BMJ *study, which began in 1960 [1], was omitted based on his unsubstantiated claim that it had "serious misclassification bias" and the 1995 study by LeVois and Layard, which also began in 1960 [40], was omitted without comment and was not even cited. However, Glantz included the other cohort studies which began in the 1960s and 1970s without any comment about their misclassification bias. Kabat and I fully addressed all these studies and the issue of misclassification bias in our 2006 meta-analysis [39]. Glantz's biased analysis regarding the relation between ETS and CHD is evident when his 2-page 2005 meta-analysis [72] is compared with our 12-page 2006 meta-analysis [39].

### Jonathan M. Samet, M.D., and the 2006 Surgeon General's Report

False and misleading statements about my research were also made by Jonathan M. Samet, M.D, M.S., who has played a prominent role in reviews of the epidemiologic evidence on ETS for over 20 years. First, Samet made a statement that neither he nor anyone else has substantiated in the May 16, 2003 Los Angeles Times, when he described my *BMJ *paper as "one very flawed study" that "just doesn't contribute" [22]. Then, he co-signed serious accusations about my research that appeared in a May 30, 2003 *BMJ *rapid response [20] and an August 30, 2003 *BMJ *letter [73]. These two items stated "Enstrom and Kabat's conclusions are not supported by the weak evidence that they offer, and although the accompanying editorial alluded to 'debate' and 'controversy', we judge the issue to be resolved scientifically, even though the 'debate' is cynically continued by the tobacco industry." To understand the outlandish nature of these accusations, recall that we used a large and highly respected dataset and accepted epidemiologic methods; we reported study details in the paper itself, in the "Prepublication History", and in our subsequent letters; we have supported our conclusions to a greater extent than can be found for any other study of ETS and mortality; our methods have never been substantively challenged; and our results are consistent with the entire body of U.S. evidence [39].

These statements from Samet might have been somewhat plausible if he had any evidence that there were errors in my 2003 paper or that I was "pro-tobacco" based on my research before 2003. But neither he nor other critics have made a plausible case for fundamental errors in my paper, and I have never been "pro-tobacco." Samet has been aware of my epidemiologic research since we both participated in the August 23–25, 1978 National Cancer Institute Workshop on "Populations at Low Risk of Cancer" held in Snowbird, Utah. The proceedings of the workshop, including the list of participants, were published in *JNCI* in November 1980 [74]. I gave three talks at this Workshop and two of them described the reduced cancer death rates among nonsmokers, one dealing with Mormons [75] and another dealing with a representative sample of U.S. nonsmokers [76]. Indeed, I have investigated the healthy lifestyles of Mormons and other nonsmokers during my entire epidemiologic career [77,78].

Further evidence of Samet's willingness to dismiss scientific evidence when it does not support his agenda appears in the June 27, 2006 release and publication of the 727-page Surgeon General's Report on "*The Health Consequences of Involuntary Exposure to Tobacco Smoke" *[79]. Samet was the Senior Scientific Editor of this report and the most influential epidemiologist involved with the report [80]. In addition, Glantz was a Contributing Editor and Thun was a Reviewer on this report. Although Samet, Thun, and Glantz were fully aware of the importance of my *BMJ *paper, as evidenced by their extensive efforts to discredit it, the paper was simply omitted from the Surgeon General's Report without comment. A search for "enstrom j" of the entire PDF version of the report [79], reveals that the only mention of the *BMJ *paper is in the Appendix on page 673, where it is listed as one of the papers not included in the report. Another search reveals that the *BMJ *paper was omitted without explanation from the database for the Report [81]. This database was prepared by Johns Hopkins University and the Centers for Disease Control and Prevention's Office on Smoking and Health. It includes "approximately 900 key articles regarding involuntary smoking and disease outcomes" and supposedly "reflects the most recent findings in the scientific literature."

In order to illustrate the selective and unscientific nature of this omission, I examined the references used in Chapters 1–10 of the Surgeon General's Report and the references in the Appendix that were not used. Of 38 total references from 2003, 33 were used in Chapters 1–10 and only 5 references, including the *BMJ *paper, were not used. Of 71 references from 2004, 53 were used and 18 were not used; of 39 references from 2005, 26 were used and 13 were not used; of 22 references from 2006, 7 were used and 15 were not used. In summary, the report used 119 references from 2003–2006, but omitted without comment the 2003 *BMJ *paper. The *BMJ *paper was the only U.S. study relating ETS to lung cancer and coronary heart disease that was omitted. Because of this omission, the Surgeon General's Report does not accurately reflect all the peer-reviewed epidemiologic evidence on the relation of ETS to lung cancer and coronary heart disease mortality in the U.S.

Chapter 7, page 423, reports: "This chapter considers the full body of evidence on secondhand smoke exposure and lung cancer published through 2002, the ending date for the systematic review of the epidemiologic studies." Based on comparing never smokers ever married to a smoker with never smokers never married to a smoker, a world wide relative risk (RR) of 1.21 (1.13–1.30) was reported on page 435. However, there is no reason for an ending date of 2002, given that other sections of the report cite results published during 2003–2006 (by my count 119 such publications are cited). It appears that the ending date of 2002 was intentionally selected in order to exclude my 2003 *BMJ *results. Consequently, the above worldwide RR is misleading because it does not reflect that fact that my results substantially weaken the U.S. evidence [1,29]. My own meta-analysis of all U.S. spousal smoking studies, yields a U.S. RR of 1.10 (1.00–1.21), which barely constitutes a relationship.

Chapter 7 contains this inaccurate statement on page 435: "There were no significant differences in the RR estimates by geographic area; the point estimate was 1.15 (95 percent CI, 1.04–1.26) for studies conducted in the United States and Canada, 1.16 (95 percent CI, 1.03–1.30) for studies conducted in Europe, and 1.43 (95 percent CI, 1.24–1.66) for studies conducted in Asia." Obviously, the RR = 1.43 for studies in Asia is statistically greater than the RR = 1.15 for studies in U.S. and Canada and the RR = 1.16 for studies in Europe. Indeed, there is substantial variation around the world and all these results cannot be accurately represented by a single RR of 1.21. This geographic variation should have been properly acknowledged in the Report.

Chapter 8 contains on page 521 selective criticism about and dismissal of the analysis by LeVois and Layard of ETS and CHD deaths in the ACS CPS I and CPS II studies [40]. This paper is important because of its size and statistical power, as discussed in our 2006 meta-analysis of ETS and CHD deaths in the U.S. [39]. One basis for the dismissal is the inaccurate statement, "The investigators did not distinguish between current exposures from spousal secondhand smoke and former exposures, nor did they separately report the effect of current spousal smoking on the risk of CHD." Table 4 of the LeVois and Layard paper clearly shows results for three levels of current ETS exposure for both males and females. Furthermore, Table 2 summarizes the dose-response relationship between ETS and CHD deaths based on the results from the three largest U.S. studies [1,40,82]. There is no meaningful difference in the results for these studies and no dose-response relationship in any of them.

Furthermore, note that the meta-analysis of ETS and CHD is summarized in Figure 8.1 on page 524. Since this figure only shows studies through 2001 it obviously omits the 2003 *BMJ *study.

The *BMJ *study has a major impact on the meta-analysis, as pointed out in our 2003 *BMJ *letter [29] and our 2006 meta-analysis [39]. Note that inclusion of *BMJ *results yields a relative risk (RR) of CHD death in the U.S. of 1.05 (0.99–1.11), based on a comparison of current to never exposure to ETS. This is much less than the summary RR (exposed/unexposed) of 1.27 (1.19–1.36) contained in Figure 8.1. The Surgeon General's Report should have pointed out that the ETS and CHD relationship is much larger outside of the U.S. than it is within the U.S. We estimated that the RR outside the U.S. is approximately 1.5 [39] and the 1999 Thun meta-analysis found the RR was 1.41 (1.21–1.65) [32]. This large difference between the RRs within the U.S. and those outside of the U.S. is worthy of further discussion and investigation, in order to determine if it is a real difference or an anomaly due to methodological issues.

The Introduction of the Surgeon General's Report makes the statement that "about 50,000 excess deaths result annually from exposure to secondhand smoke (Cal/EPA 2005). Estimated annual excess deaths for the total U.S. population are about 3,400 (a range of 3,423 to 8,866) from lung cancer, 46,000 (a range of 22,700 to 69,600) from cardiac-related illnesses, and 430 from SIDS." [79]. Given the fact that the two largest epidemiologic studies on ETS and tobacco-related mortality [1,40] have been omitted from the Surgeon General's Report and the fact that these two U.S. studies suggest a substantially weaker ETS and mortality relationship in the US, the above estimate of excess deaths appears to be an intentional exaggeration of what the entire body of scientific evidence shows. A complete evaluation of all the peer-reviewed U.S. epidemiologic evidence suggests that ETS exposure is associated with a much smaller number of lung cancer and CHD deaths in U.S. never smokers. Furthermore, there is not a "causal" relationship by traditional epidemiologic standards.

An August 23, 2006 "research news and perspective" report in *JAMA *questioned various aspects of the Surgeon General's Report, particularly findings regarding the acute effects of small amounts of ETS exposure and the claim by the Surgeon General that "There is no safe level of exposure to secondhand smoke" [83]. This *JAMA *report is particularly noteworthy because it quotes two experts who have extensive experience regarding the ETS issue. Michael Siegel, MD, MPH, a professor of social and behavioral sciences at Boston University School of Public Health and a prominent tobacco control researcher, told *JAMA *"We're really risking our credibility [as public health professionals or officials] by putting out rather absurd claims that you can be exposed briefly to secondhand smoke and you are going to come down with heart disease or cancer. People are going to look at that and say that's ridiculous." Siegel's own paper expanding on this point is published alongside the present article [84]. Furthermore, since March 2005, Siegel has posted many detailed and insightful analyses regarding ETS and tobacco control on his personal website, "The Rest of the Story: Tobacco News Analysis and Commentary" [85]. Each post includes "Comments" from readers who provide additional insights. For instance, on June 28, 2006, he posted "Surgeon General's Communications Misrepresent Findings of Report; Tobacco Control Practitioners Appear Unable to Accurately Portray the Science" [86].

John C. Bailar III, MD, PhD, a prominent epidemiologist and biostatistician, who is Professor Emeritus at the University of Chicago, told *JAMA *"It doesn't make sense for the cardiovascular risk of secondhand smoke to be as high as one third of the risk from direct smoking. . . . That's a far bigger ratio than risk for lung cancer and it's hard for me to believe that it's real" [83]. These comments are similar to those in his March 25, 1999 *NEJM *editorial on ETS and coronary heart disease, in which he stated "I regretfully conclude that we still do not know, with accuracy, how much or even whether exposure to environmental tobacco smoke increases the risk of coronary heart disease" [87]. On June 7, 2006, just 20 days before the release of the Surgeon General's Report, the Select Committee on Economic Affairs of the House of Lords in London issued an important report on the management of risk, which suggests that passive smoking in England may be a relatively minor health risk [88]. The committee obtained testimony from Professor Sir Richard Peto of the University of Oxford on February 14, 2006 [89]. Sir Richard's testimony clearly states the substantial doubt that he has about the quantitative health risks of passive smoking [90,91]. The very fact that two major reports published in the same month, June 2006, come to substantially different conclusions about the health risks of ETS indicates that these risks are still uncertain and difficult to measure accurately.

Further evidence of the uncertainty regarding the health risks of ETS is contained in the June 28, 2007 *Nature *news article on ETS. Various claims made by Glantz about the acute and chronic health effects of ETS are questioned by Peto, Bailar, and Siegel, who restated their concerns that the dangers of ETS have been exaggerated [92]. For instance, Peto stated "Passive smoking must kill some people, but the big question is how many." This statement clearly underscores the existing uncertainty and directly contradicts the June 27, 2006 statement by U.S. Surgeon General Richard H. Carmona that "The debate is over" regarding the health effects of secondhand smoke [93].

### Jonathan M. Samet, M.D., and United States of America v. Philip Morris USA, et al

One particularly pernicious aspect of the attack described above is the fact that my *BMJ *paper is now part of the largest ($280 billion) Racketeer Influenced and Corrupt Organizations Act (RICO) lawsuit ever filed, United States of America v. Philip Morris USA, et al. [Civil Action No. 99-CV-02496(GK)] [94,95]. My research and I are described in a defamatory way on pages 821–830 within the section "Defendants Used Their Jointly Controlled Organizations to Promote Their Agenda Through Symposia, Publications and a Roster of Long-time Paid Scientists" of the 2543-page pretrial "UNITED STATES' FINAL PROPOSED FINDINGS OF FACT (July 2004)" prepared by the U.S. Department of Justice (USDOJ) [96]. The trial took place in front of U.S. District Court Judge Gladys Kessler from September 2004 though June 2005 [94]. Additionally, my research and I are described in a defamatory way in several places in the 2454-page post-trial document "UNITED STATES' FINAL PROPOSED FINDINGS OF FACT (Incorporating Errata of August 16, 2005)" prepared by the USDOJ [97]. Specifically, my *BMJ *paper is listed on page vii of the Table of Contents under the category "Cooking the Books: The Manufacture of False Science to Support the Industry Position on ETS." On page 493 it is included among "examples of scientific fraud" and on page 589 it is described as "at best a contamination of the scientific literature and at worst a scientific fraud." It is discussed in detail on pages 609–615, where there are numerous false statements and distortions, such as, "the Enstrom/Kabat study is yet another self-serving, unreliable, and scientifically questionable product of the industry's unabated effort to attack the scientific consensus on passive smoking." Although no actual evidence was presented of errors in my study or of scientific misconduct on my part, the lawsuit makes it appear that I have engaged in scientific fraud.

The available evidence indicates that insertion of the *BMJ *paper was a collaborative effort of Glantz and Sharon Y. Eubanks (D.C. Bar No. 420147), Director of the USDOJ Tobacco Litigation Team from 1999 until December 2005, when she resigned from the USDOJ [98]. The following brief in Civil No. 99-CV-02496 (GK), "REPLY IN SUPPORT OF THE UNITED STATES' THIRD MOTION TO COMPEL PRODUCTION OF DOCUMENTS WITHHELD BY BROWN & WILLIAMSON BASED ON ASSERTIONS OF PRIVILEGE OR PROTECTION," was prepared by Eubanks and signed on December 5, 2003. This brief is posted on the same listserv that Glantz has used to post other defamatory information about me [99]. Pages 8, 9, and 14 of this brief contain a misleading and distorted presentation of my alleged "ties" with the tobacco industry going back "nearly 30 years." This presentation later appeared in the July 2004 and August 2005 Findings of Fact of the USDOJ lawsuit. This 2003 brief does not present any evidence challenging my honesty as a scientist or the validity of the findings in my *BMJ *paper. It is simply an attempt to smear my reputation with inappropriately constructed "ties" to the tobacco industry, based on the fact that I had correspondence with the tobacco industry regarding my epidemiologic research.

On August 17, 2006 District Court Judge Gladys Kessler issued a 1,653 page Final Opinion concluding that the tobacco industry had engaged in racketeering [100,101]. Eleven key pages from her decision, including pages discussing my study, were assembled by Glantz and posted on a UCSF website [102]. The Kessler decision includes a section entitled "The 2003 Enstrom/Kabat Study" on pages 1380–1383, as well as other references to my study. The Judge repeated in her opinion a number of the misleading and inaccurate statements about my study that are contained in the 2004 and 2005 Findings of Fact. However, the Judge identified no specific errors in the study and identified no scientific misconduct by me. At no time was I ever given an opportunity to challenge or refute the statements made about me and my research in the USDOJ Findings of Fact, in the trial itself, or in the Kessler opinion. I am now in the process of clearing my name in connection with this lawsuit and this paper represents a major step in that process. Furthermore, on October 31, 2006 the U.S. Court of Appeals of the District of Columbia Circuit granted the tobacco industry's emergency motion to stay Judge Kessler's final judgment and remedial order pending appeal [103]. On May 22, 2007 the U.S. Court of Appeals issued an order setting the briefing schedule for the appeal [104].

In formulating her comments about my study, Judge Kessler relied heavily on the testimony of Samet. On page 765 of her decision she states "Dr. Jonathan Samet, a Government expert with extraordinary qualifications, is a physician and epidemiologist with extensive experience treating patients with lung cancer and COPD." On page 1232 she states: "Dr. Samet is professor and chair of the Department of Epidemiology at the Johns Hopkins Bloomberg School of Public Health. He is also a licensed physician who is board certified in pulmonary and internal medicine. Dr. Samet is a member of the National Academy of Sciences' Institute of Medicine, the Board of Scientific Counselors of the National Cancer Institute, and EPA's Clean Air Scientific Advisory Committee. He is a recipient of the Surgeon General's Medallion and has participated as an author and/or editor of nine Surgeon General's Reports, including as Consulting Scientific Editor and author for the 1986 Report. He has participated in four NCI monographs in its series on smoking and health. He chaired the 2002 review of active and passive smoking and health for the International Agency for Research on Cancer of the World Health Organization. . . . after considering Dr. Samet's superb academic credentials, his vast experience working on Surgeon General Reports and NCI monographs, his continuing practice of medicine, as well as his demeanor and responsiveness to cross-examination, the Court fully credits his testimony." On page 1234 she states: "The Court accepts and credits Dr. Samet's conclusions, based on his expertise, as well as the other factual findings herein, that exposure to secondhand smoke causes lung cancer and coronary heart disease in adults and a number of respiratory diseases in children."

It is worth repeating the allegations in the Kessler decision, first to point out that they are the same false and misleading claims about the Enstrom/Kabat study by the ACS, Samet, Glantz, and others that are described above, and second to show how obviously incorrect they are. The Enstrom/Kabat study was not "CIAR-funded and managed" and was not "funded and managed by the tobacco industry through CIAR and Philip Morris." Although the study was partially funded by CIAR, it was not managed by either CIAR or Philip Morris. Indeed, CIAR assigned its entire award for the study to UCLA in 1999 just before CIAR was dissolved as a condition of the Master Settlement Agreement [105]. CIAR did not even exist when my study was being completed. The study was conducted and published without any influence from the tobacco industry. The claim that the "American Cancer Society had repeatedly warned Enstrom that using its CPS-I data in the manner he was using it would lead to unreliable results" is utterly false and the ACS has produced no documentation to support this claim. The claim "Enstrom and Kabat's conclusions are not supported by the weak evidence that they offer" made by Samet and others is utterly false because our conclusions are fully supported by the evidence in our *BMJ *paper, as stated earlier.

In addition, Samet made an inaccurate and incomplete statement in his Written Direct testimony of September 20, 2004 (page 184, lines 8–9): "When the 2002 meta-analysis carried out by IARC was redone in 2004 to include this [Enstrom and Kabat] study, the positive findings were unchanged." [106]. This statement is inaccurate because the August 30, 2003 *BMJ *letter signed by Samet correctly states: "Adding the result from Enstrom and Kabat to the IARC analysis reduces the pooled estimate to 1.23." [73]. In addition, this statement is incomplete because Samet failed to state that the Enstrom and Kabat results reduced the pooled risk ratio estimates for U.S. studies to about 1.10 for lung cancer and to about 1.05 for coronary heart disease [39]. The Enstrom/Kabat summary risk ratios are far below the widely stated summary risk ratios of about 1.25 and are not consistent with the estimate that "about 50,000 excess deaths results annually from exposure to secondhand smoke" in the US, as stated on page 8 of the Surgeon General's Report [79].

Samet made a false statement in this September 20, 2004 testimony when he claimed (page 192, lines 21–23): "Except for the analyses of CPS I and CPS II presented by LeVois and Layard in 1995, all other studies have demonstrated at least a modest increase in risk for fatal and nonfatal CHD due to secondhand smoke exposure." [106]. Our *BMJ *study showed no increase in risk for fatal CHD, other than the insignificant statistical fluctuation that was also present in the LeVois and Layard paper, and reference to our study should have been included in Samet's testimony.

Since no errors had been found in our paper, and since Kabat and I had clearly declared there was no tobacco industry influence on our results (and no one has found any evidence to the contrary), our research did not warrant inclusion in the USDOJ lawsuit. The citation of our study in the Kessler decision appears to be primarily due to the false and misleading statements about our research made by Samet. All of this casts doubt on the ability of Samet to be objective regarding the subject of ETS.

Further evidence of Samet's campaign against me appeared in the May 4, 2007 *Chronicle of Higher Education *as a two-page, 15-inch by 22-inch advertisement "Why do the University of California Regents still cash checks from tobacco racketeers?" [107]. This advertisement by "Campaign to Defend Academic Integrity" [108] is an appeal to UC Regents to implement a tobacco funding ban and it makes direct reference to me and my tobacco industry funding. Statements throughout the advertisement falsely characterize me and my research: "To make vivid how Big Tobacco co-opted world-class research institutions for its disinformation and legal defense strategies, the Court cited the misuse of American Cancer Society data by a non-faculty researcher at UCLA. . . Big Tobacco's investment in UCLA bought it the chance to argue falsely, using UCLA's name, that the science on secondhand smoke was inconclusive, to battle public health measures. Whatever the tobacco industry gains from the University, the University loses. The public loses, too." This compounding of the defamation in the court papers through paid advertising was signed by 21 prominent individuals who identify themselves as "among those who support action by the University of California Regents to refuse all future tobacco industry funding." The signatories include both Samet and Eubanks, who obviously have been directly involved in lobbying the UC Regents, a position that compromises their objectivity with regard to my inclusion in the USDOJ lawsuit. Given the obsessive focus on my tobacco industry funding, it is noteworthy that there is no indication of the funding and competing interests of those associated with this advertisement. The *Chronicle of Higher Education *website states that a "tabloid-page spread" advertisement like this one costs $22,630 [109], a sum unlikely to have been paid by the signatories themselves.

Based on the record presented above, Eubanks has obviously dealt extensively with both Glantz and Samet regarding the issue of my *BMJ *paper and the USDOJ lawsuit. She injected herself directly into the UC tobacco industry funding ban issue with a lecture before the Regents on July 18, 2007, when she described the USDOJ lawsuit and its connection to UC [110]. She claimed that Judge Kessler was "a neutral fact finder, a federal judge, who made her findings of conspiratorial conduct objectively" based on "a full and fair record." However, she knows that the record is not objective and that I was never given any opportunity to defend myself and my *BMJ *paper during the trial. In an eloquent defense of academic freedom at UC, the 2006–2007 UC Academic Senate Chair John B. Oakley challenged Eubank's linkage of the USDOJ lawsuit to UC and raised the issue of whether Judge Kessler's opinion would ultimately be upheld upon appeal [11188d]. A clearer understanding of this entire issue can be gained by carefully listening to the Eubanks and Oakley audio files [110,111].

### Jonathan M. Samet, M.D., and Conflict of Interest

Samet has not revealed his competing interests on the subject of ETS as they relate to the *BMJ *rr [20], the *BMJ *letter [73], the IARC Report [50], the *JNCI *article [52], the Surgeon General's Report [79], his USDOJ lawsuit testimony [106], or the Chronicle of Higher Education advertisement [107]. Given that Samet has criticized persons who disagree with his views on ETS because of their competing interests, it is fair and reasonable to ask why he has failed to report his own substantial competing interests. A careful examination of the Surgeon General's Report reveals that it contains no conflict of interest disclosures for Senior Scientific Editor Samet or for any of the other editors or reviewers. In addition, an examination of the other items above reveals the Samet has not disclosed a financial conflict of interest which could have compromised his objectivity on ETS. This imbalance further suggests that the attacks on my research have nothing to do with a principled concern about conflicts of interest, but are purely a matter of not liking the results.

The article, "smoke out!", in the Spring 2003 issue of *Johns Hopkins Public Health*, "The Magazine of the Johns Hopkins Bloomberg School of Public Health" [112] reveals that, "After three years of preparation, Samet testified in the landmark 1998 Minnesota tobacco trial that smoking causes certain diseases like lung cancer" and that Samet was "working on the federal government's $289 billion lawsuit that accuses tobacco companies of 50 years of deceptive marketing," which is the USDOJ lawsuit discussed above. Later, the article stated "In March, the Flight Attendant Medical Research Institute honored Samet with the '...Dr. William Cahan Distinguished Professor' Award and $600,000 over 3 years to combat tobacco-related disease."

According to the Flight Attendant Medical Research Institute (FAMRI) website, the 'Dr. William Cahan Distinguished Professor' award to Samet during 2003–2006 was "made in recognition of the recipients' ongoing work in combating the diseases caused by exposure to second hand tobacco smoke" [113]. In addition, Samet has a prominent role in the current multi-million dollar Johns Hopkins FAMRI Center of Excellence [114]. This Center was established in 2005 and currently has 30 FAMRI-funded research projects on "diseases and medical conditions caused from exposure to tobacco smoke," including one by Samet on "Reducing the Risks of Secondhand Tobacco Smoke Globally" [113].

FAMRI is a foundation established as a result of an October 1991 Class Action suit filed in Miami's Dade County Circuit Court in Florida, known as *Broin v. Philip Morris *[116]. This suit was filed against the tobacco industry on behalf of flight attendants who sought damages for diseases and deaths allegedly caused by their exposure to second hand tobacco smoke in airline cabins [117]. A settlement was reached in October 1997 between the plaintiffs and four tobacco companies. The Settlement Agreement included the establishment of a not-for-profit medical research foundation with funding by the tobacco industry of $300 million. The Foundation was to have no tobacco company involvement, other than funding. The purpose of the foundation was "to sponsor scientific research with respect to the early detection and cure of diseases associated with cigarette smoking" [118]. FAMRI, as it was actually established, has a distinctly different mission, which is "to sponsor scientific and medical research for the early detection, prevention, treatment and cure of diseases and medical conditions caused from exposure to tobacco smoke." [117]. Since FAMRI's mission statement assumes that diseases like lung cancer and CHD are caused by "exposure to tobacco smoke," this funding source may have influenced Samet's decisions about which epidemiologic studies he chooses to believe and which ones he chooses to ignore, and thus should have been disclosed. As noted in an August 23, 2006 *JAMA *editorial, in published articles it is important "that readers are aware of the authors' financial relationships and potential conflicts of interest so that these readers can interpret the article in light of that information" [119].

### Jonathan M. Samet, M.D., and the 1992 EPA Report

One might wonder how omissions, distortions, and exaggerations like those pointed out above could occur in a document as important as a Surgeon General's Report on ETS. To better understand this phenomena one must realize that Samet has dealt with the ETS issue in this manner for many years. In particular, he played a major role in the epidemiologic analysis for the December 1992 report on * Health Effects of Passive Smoking: Lung Cancer and Other Disorders: The Report of the United States Environmental Protection Agency *[120]. This EPA report classified ETS as a Group A human carcinogen, which causes about 3,000 lung cancer deaths per year in the U.S. The findings from this report were used in the *Broin v. Philip Morris *litigation described above.

The epidemiologic methodology and conclusions of the EPA report have been severely criticized. One of the harshest critiques is the 92-page Decision issued by Federal Judge William L. Osteen on July 17, 1998, which overturned the report in the U.S. District Court [121]. For instance, in his conclusion Judge Osteen wrote: "In conducting the Assessment, EPA deemed it biologically plausible that ETS was a carcinogen. EPA's theory was premised on the similarities between MS [mainstream smoke], SS [sidestream smoke], and ETS. In other chapters, the Agency used MS and ETS dissimilarities to justify methodology. Recognizing problems, EPA attempted to confirm the theory with epidemiologic studies. After choosing a portion of the studies, EPA did not find a statistically significant association. EPA then claimed the bioplausibility theory, renominated the a priori hypothesis, justified a more lenient methodology. With a new methodology, EPA demonstrated from the 88 selected studies a very low relative risk for lung cancer based on ETS exposure. Based on its original theory and the weak evidence of association, EPA concluded the evidence showed a causal relationship between cancer and ETS. The administrative record contains glaring deficiencies. . . ."

In order to more fully understand the EPA report and its inherent flaws, one must read the complete Osteen decision [121], as well as the books *Passive Smoke: The EPA's Betrayal of Science and Policy *by Drs. Gio B. Gori and John C. Luik [122], *Ashes to Ashes: America's Hundred-Year Cigarette War, the Public Health, and the Unabashed Triumph of Philip Morris *by Richard Kluger [123], *For Your Own Good: The Anti-Smoking Crusade and the Tyranny of Public Health *by Jacob Sullum [124], and the *Brill's Content *magazine article "Warning: Secondhand Smoke May NOT Kill You" by Nicholas Varchaver [125]. Finally, one must read the January 28, 1993 *Investors' Business Daily *article "Is EPA Blowing Its Own Smoke? How Much Science Is Behind Its Tobacco Finding?" by Michael Fumento, who stimulated my own interest in the ETS issue [126].

### 2006 Congress of Epidemiology and Trofim Denisovich Lysenko Analogy

In order to explain the phenomenon that has made this defense of my epidemiologic research necessary, Geoffrey Kabat, Sheldon Ungar, and I presented a symposium entitled "Reassessment of the Long-term Mortality Risks of Active and Passive Smoking" at the 2^nd ^North American Congress of Epidemiology in Seattle, Washington on June 24, 2006 [127]. We described major misrepresentations that are currently occurring with regard to the epidemiology of both active and passive smoking, as well as the silencing of science associated with this area of epidemiology. I presented the rationale for the symposium based on the fact that important epidemiologic findings have been ignored or mischaracterized in prior assessments. Then I presented evidence that the adverse effects of active smoking on mortality are less reversible by cessation than generally believed, based on randomized controlled trials involving smoking cessation and "natural experiments" involving the CA CPS I cohort and several other cohorts [31,128,129]. Kabat presented evidence that the relationship between passive smoking and mortality is weaker than generally believed, particularly within the United States, based on our two recent ETS papers [1,39]. Ungar described the "silencing of science" phenomenon with regard to our May 17, 2003 *BMJ *paper that he documented and described in his 2005 paper [27].

In this symposium we addressed several important issues: 1) the implications of our reassessment for the relative dangers of active and passive smoking; 2) the way in which ideological and political agendas have influenced the interpretation of epidemiologic evidence; and 3) the importance of separating non-scientific agendas from objective assessment of evidence. We made the case that: 1) all epidemiologic findings must be evaluated in a fair and consistent manner in order to obtain an accurate assessment of the mortality risks of active and passive smoking; 2) epidemiologic findings must be judged on their merits and not on extraneous factors; and 3) additional epidemiologic research in this area needs to be conducted free of partisanship. Our complete presentations are available on the Scientific Integrity Institute website [130], and they include our PowerPoint slides and the audio files for our lectures.

It is quite informative to compare our Symposium with the June 23, 2006 lecture "Using Epidemiologic Evidence to Advance Health: Dealing with Critics and Criticisms" given by Samet at the same Congress of Epidemiology [131]. Samet discussed the use of epidemiologic evidence in public health policy making with regard to the environmental epidemiology issues in which he has been involved. In particular, he discussed the epidemiologic evidence on the relationship between passive smoking and lung cancer just four days before the June 27, 2006 release of the Surgeon General's Report on involuntary smoking for which he was Senior Scientific Editor [79]. He talked about the criticism of weak epidemiologic relationships, such as those described in major documents like the 2006 Surgeon General's Report. But he failed to mention that much of this criticism is due to the fact that he has attempted to turn weak and inconsistent observational epidemiologic evidence into an undisputed causal relationship. He talked about how critics raise epidemiologic issues like confounding and bias, but he failed to acknowledge his own biased presentation of the evidence, including omitting my *BMJ *paper from the report and failing to acknowledge that the U.S. evidence is weaker than the evidence outside of the U.S.

Also, it is quite telling how Samet dismissed critics of the causal relationship between passive smoking and lung cancer by classifying them as "stakeholders" linked with the "tobacco industry." He implied that it is not necessary to address the merits of their criticisms simply because they are stakeholders in decisions related to passive smoking. However, he failed to disclose his own financial interests that surely put him in the stakeholder category. He certainly never mentions that his FAMRI money originates from the tobacco industry, making it remarkably similar to my CIAR funding. Samet's lecture provides insight into his thought processes and the ways in which he manipulates evidence to fit his vision of an epidemiologic relationship with public policy implications. The transcript of a key portion of his lecture is available [132], as is the audio file [133].

We concluded our Symposium by drawing an analogy between the current situation involving ETS epidemiology in the United States and the historical situation involving agronomist Trofim Denisovich Lysenko and plant genetics in the Soviet Union during the period of 1927–1962 [2]. While it is common to invoke George Orwell or Joseph McCarthy in discussions like this, I believe the lessons from the admittedly more extreme Lysenko case are more analogous and informative. Although ETS epidemiologic evidence has never been conclusive, several major reports have been issued with definitive conclusions about a "causal relationship" between ETS and mortality. All major U.S. government and private health agencies have declared that a causal relationship exists and these organizations have created "a regime of truth that cannot be intelligibly questioned." These organizations then use any means necessary to enforce this "regime of truth." Since the publication of the influential null findings in my *BMJ *paper, which contradict the "regime of truth," I have been subjected to a massive *ad hominem *attack, my career has been threatened, and my paper has been dismissed because of its politically incorrect findings. In addition, I was inserted into a massive lawsuit by my own government in a manner that makes it appear that I have committed "scientific fraud" and have been engaged in racketeering with the tobacco industry. There also has been the attempt to force the University of California to ban the tobacco industry funding that I have used and to restrict future research in the areas of tobacco-related diseases that I have been investigating.

Lysenko used his influence and backing by the Soviet government to create a "regime of truth" and to stop others' research in order to promote scientifically invalid "vernalization" and Lamarckian plant genetics. He was also successful in attacking and destroying his critics, like Nicolai Vavilov, who espoused proper Mendelian plant genetics. Because Lysenko prevailed for such a long period of time, crop yields were low, Soviet agriculture regressed, and Soviet citizens suffered greatly and many faced starvation. During this same period, proper plant genetics were developed and implemented in the U.S. and this resulted in the greatly increased crop yields that have made U.S. food production so incredibly successful. The entire saga of "Lysenko pseudoscience" has been extensively described in websites about Lysenko [134], journal articles [2,135], and books [136-138].

Prominent U.S. epidemiologists and activists are wielding governmental influence to distort the epidemiology of both active and passive smoking in the U.S. and are contributing to a Lysenko-like research environment where it is virtually impossible to conduct research that produces politically incorrect findings, such as, those in my *BMJ *paper. Much additional research is needed because the primary tobacco-related disease, lung cancer, still causes 160,000 deaths per year in the U.S. and will not go away any time soon. This Lysenko-like research environment needs to end and epidemiologists must be free to conduct additional research on tobacco-related diseases with a variety of funding sources without fear of the kind of attacks that I have experienced.

### A Challenge to ACS and Michael J. Thun, M.D

Some of the controversy about the relation of ETS and tobacco-related mortality in the largest U.S. observational epidemiologic studies could be settled if Thun fully, fairly, and transparently analyses the CPS I and CPS II cohort data that the ACS currently possesses. Because of their size and length of mortality follow-up, these two cohorts contain the vast majority of the potentially available U.S. evidence on ETS, and are already the basis for important U.S. evidence on active smoking. Given the epidemiologic expertise of Thun and the availability of the appropriate CPS I and CPS II data, such an analysis could be conducted in a matter of weeks. In the interest of better understanding cancer etiology, the ACS should fully analyze these important data. I have provided sample Tables 3, 4, 5, 6 and 7 so that Thun can present results that are directly comparable to those presented in my *BMJ *paper [1].

**Table 3:** Level of spousal smoking related to deaths from lung cancer and coronary heart disease among never smokers in California CPS I cohort as of 1959. Relative risk (RR with 95% CI) comparing persons with each level of exposure to those without exposure. Proportional hazards linear model (PHREG) is used and RR is adjusted for age at entry. Results shown are from Tables 7 and 8 of 2003 *BMJ *paper [1]. See note below.*

		Lung cancer (ICD7 = 162–3, ICD8&9 = 162)				Coronary heart disease(ICD7 = 420, ICD8&9 = 410–4)			
			
		1 Jan 60–30 Sep 72		1 Jan 60–31 Dec 98		1 Jan 60–30 Dec 72		1 Jan 60–31 Dec 98	
Spousal smoking (ETS index level) as of 1959	Subjects	Deaths	Age-adjusted RR (95% CI)	Deaths	Age-adjusted RR (95% CI)	Deaths	Age-adjusted RR (95% CI)	Deaths	Age-adjusted RR (95% CI)
					
	nn,nnn	nnn	x.xx (x.xx-x.xx)	nnn	x.xx (x.xx-x.xx)	nnn	x.xx (x.xx-x.xx)	nnn	x.xx (x.xx-x.xx)
Males									
Never (1)	7,458		1.00	65	1.00			1,860	1.00
Former (2)	624			5	0.92 (0.37–2.30)			126	0.94 (0.78–1.12)
Current									
1–9 cpd (3)	392							81	0.97 (0.78–1.21)
10–19' cpd(4)	513							99	0.86 (0.70–1.05)
20 cpd (5)	458							81	0.92 (0.74–1.15)
21–39 cpd (6)	129							27	1.16 (0.79–1.69)
40+ cpd (7)	45							13	1.29 (0.75–2.22)
Current – total	1,537			9	0.69 (0.34–1.39)			301	0.94 (0.83–1.07)
Ever	2,161			14	0.75 (0.42–1.35)			427	0.94 (0.85–1.05)
									
Females									
Never (1)	7,399		1.00	51	1.00		1.00	1,053	1.00
Former (2)	6,858			51	1.08 (0.73–1.60)			1,059	1.02 (0.93–1.11)
Current									
Pipe/cigar (3)	2,691							389	0.99 (0.88–1.11)
1–9 cpd (4)	1,102							183	1.13 (0.97–1.33)
10–19 cpd (5)	2,117							310	1.03 (0.91–1.17)
20 cpd (6)	3,288							412	1.04 (0.92–1.16)
21–39 cpd (7)	1,646							167	0.95 (0.80–1.12)
40+ cpd (8)	841							72	0.83 (0.65–1.06)
Current – total	11,685			75	0.93 (0.65–1.33)			1,533	1.01 (0.93–1.09)
Ever	18,543			126	0.99 (0.72–1.37)			2,592	1.01 (0.94–1.08)

*Tables C–G should be completed by Michael J. Thun, M.D., of the ACS to fully present results on ETS and lung cancer and coronary heart disease mortality in the CPS I cohort during 1960–1972 and in the CPS II cohort during 1982–1998 in a format that is the same as that used for the 1960–1998 CA CPS I results in the 2003 *BMJ *paper (1), some of which are shown in Table C.

**Table 4:** Level of spousal smoking related to deaths from lung cancer and coronary heart disease among never smokers in 25-state CPS I cohort as of 1959. Relative risk (RR with 95% CI) comparing persons with each level of exposure to those without exposure. Proportional hazards linear model (PHREG) is used and RR is adjusted for age at entry.

		Lung cancer (ICD7 = 162–3, ICD8 = 162)				Coronary heart disease (ICD7 = 420, ICD8 = 410–4).			
			
		1 Jan 60–31 Dec 65		1 Jan 60–30 Sep 72		1 Jan 60–31 Dec 65		1 Jan 60–30 Sep 72	
Spousal smoking (ETS index level) as of 1959	Subjects	Deaths	Age-adjusted RR (95% CI)	Deaths	Age-adjusted RR (95% CI)	Deaths	Age-adjusted RR (95% CI)	Deaths	Age-adjusted RR (95% CI)
					
	nn,nnn	nnn	x.xx (x.xx-x.xx)	nnn	x.xx (x.xx-x.xx)	nnn	x.xx (x.xx-x.xx)	nnn	x.xx (x.xx-x.xx)
Males									
Never (1)			1.00		1.00		1.00		1.00
Former (2)									
Current									
1–9 cpd (3)									
10–19 cpd(4)									
20 cpd (5)									
21–39 cpd (6)									
40+ cpd (7)									
Current – total									
Ever									
									
Females									
Never (1)			1.00		1.00		1.00		1.00
Former (2)									
Current									
Pipe/cigar (3)									
1–9 cpd (4)									
10–19 cpd (5)									
20 cpd (6)									
21–39 cpd (7)									
40+ cpd (8)									
Current – total									
Ever									

**Table 5:** Level of spousal smoking related to deaths from lung cancer and coronary heart disease among never smokers in CPS II cohort as of 1982. Relative risk (RR with 95% CI) comparing persons with each level of exposure to those without exposure. Proportional hazards linear model (PHREG) is used and RR is adjusted for age at entry.

		Lung cancer (ICD9 = 162)				Coronary heart disease (ICD9 = 410–4)			
			
		1 Sep 82–31 Dec 89		1 Sep 82–31 Dec 98		1 Sep 82–31 Dec 89		1 Sep 82–31 Dec 98	
Spousal smoking (ETS index level) as of 1982	Subjects	Deaths	Age-adjusted RR (95% CI)	Deaths	Age-adjusted RR (95% CI)	Deaths	Age-adjusted RR (95% CI)	Deaths	Age-adjusted RR (95% CI)
					
	nn,nnn	nnn	x.xx (x.xx-x.xx)	nnn	x.xx (x.xx-x.xx)	nnn	x.xx (x.xx-x.xx)	nnn	x.xx (x.xx-x.xx)
Males									
Never (1)			1.00		1.00		1.00		1.00
Former (2)									
Current									
1–9 cpd (3)									
10–19 cpd(4)									
20 cpd (5)									
21–39 cpd (6)									
40+ cpd (7)									
Current – total									
Ever									
									
Females									
Never (1)			1.00		1.00		1.00		1.00
Former (2)									
Current									
Pipe/cigar (3)									
1–9 cpd (4)									
10–19 cpd (5)									
20 cpd (6)									
21–39 cpd (7)									
40+ cpd (8)									
Current – total									
Ever									

**Table 6:** Total self-reported hours of ETS exposure per day related to deaths from lung cancer and coronary heart disease among all never smokers in CPS II cohort with data on self-reported ETS exposure as of 1982. Relative risk (RR with 95% CI) comparing persons with each level of exposure to those without exposure. Proportional hazards linear model (PHREG) is used and adjusted for age at entry.

		Lung cancer (ICD9 = 162)				Coronary heart disease (ICD9 = 410–4)			
			
		1 Sep 82–31 Dec 89		1 Sep 82–31 Dec 98		1 Sep 82–31 Dec 89		1 Sep 82–31 Dec 98	
Total daily hours of ETS exposure as of 1982	Subjects	Deaths	Age-adjusted RR (95% CI)	Deaths	Age-adjusted RR (95% CI)	Deaths	Age-adjusted RR (95% CI)	Deaths	Age-adjusted RR (95% CI)
					
	nn,nnn	nnn	x.xx (x.xx-x.xx)	nnn	x.xx (x.xx-x.xx)	nnn	x.xx (x.xx-x.xx)	nnn	x.xx (x.xx-x.xx)
Males									
0 hours			1.00		1.00		1.00		1.00
1									
2									
3									
4									
5									
6									
7									
8+									
Current total (1+)									
									
Females									
0 hours			1.00		1.00		1.00		1.00
1									
2									
3									
4									
5									
6									
7									
8+									
Current total (1+)									

**Table 7:** 1982 level of spousal smoking related to total self-reported ETS exposure among never smokers in 1982 CPS II cohort and 1992 CPS II Nutrition cohort.

		Percent distribution of 1982 total daily hours of ETS exposure						Percent distribution of 1992 total weekly hours of ETS exposure				
				
Spousal smoking as of 1982	1982 subjects	0	1	2	3–7	8+	1992 subjects	0	1–7	8–14	15–49	50+
				
Males												
Never (1)												
Former (2)												
Current												
1–9 cpd (3)												
10–19 cpd(4)												
20 cpd (5)												
21–39 cpd (6)												
40+ cpd (7)												
Current – total												
Ever												
												
Females												
Never (1)												
Former (2)												
Current												
Pipe/cigar (3)												
1–9 cpd (4)												
10–19 cpd (5)												
20 cpd (6)												
21–39 cpd (7)												
40+ cpd (8)												
Current – total												
Ever												

In addition, Thun should analyze the CPS II cohort as a "natural experiment" of smoking cessation and mortality trends in a manner similar to what I have done. Such an analysis would test my hypothesis, based on analysis of the CA CPS I and three other U.S. cohorts, that the long-term adverse mortality effects of active smoking are more dangerous than generally believed because they are less reversible by cessation than generally believed [31,128,129]. The ACS owes it to the over two million Americans who are subjects in the CPS I and CPS II cohorts, as well as to those Americans who support the ACS, to produce epidemiologic findings that accurately and completely describe the mortality risks of active and passive smoking in their data.

In order to determine if a full analysis of ETS and mortality in the CPS II cohort supports the analysis of ETS and mortality in the CA CPS I cohort presented in my *BMJ *paper, I sent Thun a June 21, 2007 email request that he complete Tables 5, 6 and 7. Thun replied with a June 26, 2007 letter in which he gave several reasons why he would not complete Tables 5, 6 and 7. He stated "In summary, I do not believe that the analyses you request in CPS-II would produce scientifically meaningful results" [139]. He indicated no willingness to do further CPS II analyses of any kind, even analyses of the relationship of ETS to mortality during the past fifteen years. This is the latest evidence supporting the extensive "silencing of science" phenomena that currently exists with regard to ETS epidemiology in the U.S.

To illustrate the existing bias in the release of ACS results, it is quite informative to note the response by Thun to the September 26, 1994 letter that he received from Glantz [140], regarding the CPS II analyses that LeVois and Layard conducted in 1994 and published in 1995 [40]. Thun sent Glantz a detailed November 4, 1994 letter which included preliminary CPS II analyses and criticisms and described plans to do further CPS II analyses [141]. Responses to Thun's CPS II analyses and criticisms were then made by LeVois [142] and Layard [143]. All of this correspondence and commentary reinforces the continuing need for a full and objective analysis of the CPS I and CPS II data possessed by ACS.

## Conclusion

It is very disturbing that a major health organization like the ACS has made false and misleading statements about me and my May 17, 2003 *BMJ *paper for over four years. It is further disturbing that prominent individuals like Thun, Samet, and Glantz have continued to attack the findings in the *BMJ *paper, even though I have presented extensive evidence that supports the validity of these findings. In addition, it is reprehensible that the *BMJ *paper was inserted in the USDOJ RICO lawsuit and omitted from the 2006 Surgeon General's Report. These actions must be kept in mind when evaluating the honesty, integrity, and objectivity of those responsible.

These criticisms may sound personally defensive, and indeed when one is so personally attacked, some personal defense is necessary. But this is also a defense against epidemiology becoming "Lysenko pseudoscience," where the validity of methods and studies is based merely on those results that are preferred by influential advocates and researchers and contrary results are discredited using the tactics of Lysenko. Epidemiologic science is not inherently pseudoscience, of course, but the process that has led to many current claims about ETS is.

Hopefully, epidemiology can continue as a field in which all legitimate research findings can be published and objectively evaluated, including those findings considered to be controversial. However, this will happen only if advocacy organizations like the ACS and activists like Glantz refrain from unethically smearing honest scientists and putting out false and misleading statements. In addition, epidemiologists like Thun must honestly analyze all the epidemiologic evidence that they possess and fully report their results, and epidemiologists like Samet must not omit important and accurate research findings from a major document such as the Surgeon General's Report. Such omissions and actions have seriously distorted the evidence on the health effects of ETS exposure, particularly within the US.

Hopefully, this entire episode will help prevent similar episodes in the future. Furthermore, this episode will be particularly valuable if it eventually leads to a full and objective analysis of the important epidemiologic evidence that the ACS possesses on both active and passive smoking. In the meantime, epidemiologists and others interested in a full assessment of the available epidemiologic evidence on the health effects of ETS should carefully read and study this document and all the references and tables that are included in it.

## Competing interests

Funding of this paper is the same as that of reference 39. The content of this paper is based on the knowledge I have acquired during my entire epidemiologic career, during which I have had many funding sources. My competing interests are fully discussed in the text of this paper and in reference 1 and are known worldwide thanks largely to the efforts of Glantz, Thun, and Samet. My personal stake in the matters discussed here should be self-evident. In order to address concerns about my competing interests, this paper is entirely transparent and its contents can be verified with the references cited.
